# *MDM2* promoter polymorphism del1518 (rs3730485) and its impact on endometrial and ovarian cancer risk

**DOI:** 10.1186/s12885-017-3094-y

**Published:** 2017-02-03

**Authors:** Liv B. Gansmo, Merete Bjørnslett, Mari Kyllesø Halle, Helga B. Salvesen, Pål Romundstad, Kristian Hveem, Lars Vatten, Anne Dørum, Per E. Lønning, Stian Knappskog

**Affiliations:** 1 Section of Oncology, Department of Clinical Science, University of Bergen, Haukeland University Hospiltal, Jonas Lies veg 87, 5021 Bergen, Norway; 2 Department of Oncology, Haukeland University Hospital, Bergen, Norway; 3 Department of Public Health, Faculty of Medicine, Norwegian University of Science and Technology, Trondheim, Norway; 4 Department of Molecular Oncology, Oslo University Hospital Radium Hospitalet, Oslo, Norway; 5 Institute for Cancer Research, University of Oslo, Oslo, Norway; 6 Department of Gynecology and Obstetrics, Haukeland University Hospital, Bergen, Norway; 7 Centre for Cancer Biomarkers (CCBIO), Department of Clinical Science, University of Bergen, Bergen, Norway; 8 Department of Gynecologic Oncology, Oslo University Hospital, Norwegian Radium Hospital, Oslo, Norway

**Keywords:** MDM2, Del1518, Cancer risk, Ovarian cancer, Endometrial cancer

## Abstract

**Background:**

The del1518 (rs3730485) polymorphism is an in/del variant in the *MDM2* promoter P1. The variant is in complete linkage disequilibrium with *MDM2* SNP309 (rs2279744) and has previously been found associated with an increased risk of colon cancer. In this study we assessed the impact of *MDM2* del1518 on risk of ovarian and endometrial cancer.

**Methods:**

Here, we genotyped del1518 in two large hospital-based series of patients diagnosed with ovarian (*n* = 1,385) or endometrial (*n* = 1,404) cancer and performed risk estimations as compared to the genotype distribution among 1,872 healthy female controls.

**Results:**

In overall analysis we observed no association between del1518 and risk of either ovarian or endometrial cancer. However, stratifying according to SNP309 status, we found the del1518 variant to be associated with a reduced risk of endometrial cancer among individuals carrying the SNP309TT genotype both in the dominant (OR = 0.64; 95% CI = 0.45 – 0.90) and the recessive model (OR = 0.80; 95% CI = 0.65 – 1.00). No such association was observed for ovarian cancer risk.

**Conclusion:**

We found the *MDM2* del1518 del variant to be associated with reduced risk of endometrial cancer among individuals carrying the *MDM2* SNP309TT genotype.

**Electronic supplementary material:**

The online version of this article (doi:10.1186/s12885-017-3094-y) contains supplementary material, which is available to authorized users.

## Background

The protein product of the Mouse Double Minute 2 homolog (*MDM2*) gene is one of the main regulators of the tumor suppressor p53. MDM2 inhibits p53, not only by direct binding, but also by directing it to proteasomal degradation. p53, on the other hand, induces *MDM2* transcription in response to genotoxic stress [1–5]. Increased levels of MDM2, through mechanisms such as gene amplification, increased transcription and elevated translation has been observed in several human cancers [6–8], and *MDM2* overexpression has been suggested to be an alternative mechanism of p53 inactivation and tumor promotion [8, 9].

Over the last decade, single nucleotide polymorphisms (SNPs) in the *MDM2* promoter regions have been reported to affect *MDM2* transcription [10–12]. The most studied *MDM2* SNP, SNP309T > G (rs2279744) was found to increase the binding affinity between the *MDM2* promoter 2 region and the transcription factor Sp1 (specificity protein 1), resulting in increased MDM2 mRNA and protein levels [10]. While initial reports indicated the SNP309G allele to be associated with enhanced risk and early diagnoses of several tumor forms, subsequent data have been at conflict, indicating potential gender as well as ethnic differences [13–20]. A potential reason for these findings may be interaction with other SNP variants located in the *MDM2* promoter areas.

Previously, we and others reported a novel *MDM2* SNP located 24 base pairs upstream from SNP309; SNP285G > C (rs117039649) that also affect Sp1 binding to the *MDM2* promoter [11, 21, 22]. Notably, this SNP was found to be differentially distributed across different ethnic groups [11, 23].

Contrasting SNP309 and SNP285, which both are located in the *MDM2* promoter P2, the 40 bps insertion/deletion polymorphism del1518 (rs3730485) is located in the *MDM2* promoter P1. The del1518 variant has been reported to be in linkage disequilibrium (LD) with SNP309, forming a SNP309T/del1518 del haplotype [24, 25]. While the del variant has been associated with decreased *MDM2* expression [12], results so far have linked the del1518 del allele to an increased risk of hepatocellular carcinoma, colon cancer and uterine leiomyoma [25–27] but with no associations to epithelial ovarian cancer, esophageal squamous cell carcinoma, breast, lung or prostate cancer risk [24, 25, 28–30].

In the present study we assessed the potential association between del1518 and risk for ovarian and endometrial cancer in large hospital based sample sets, previously genotyped for SNP309 and SNP285, enabling assessment of potential synergisms between the three SNPs.

## Methods

### Study populations

All cases included in this case–control study were obtained from hospital-based cohorts of Norwegian patients of whom the great majority were Caucasians diagnosed with endometrial (*n* = 1,404) and ovarian cancer (*n* = 1,385) described in detail previously [31]. For comparison, we used the previously reported genotypes of the female fraction (*n* = 1,872) from a sample set of 3,749 healthy Norwegian individuals [32]. These individuals were initially drawn from the population based Cohort of Norway (CONOR) study [33].

For the endometrial cancer samples we had histological status for 1,320 and for the ovarian cancer samples histopathological status was available for 1,071 [31].

### *MDM2* genotyping

All samples were genotyped for *MDM2* del1518 by using DNA extracted from white blood cells as previously described [25]. Briefly, the region of *MDM2* containing the del1518 indel polymorphism was amplified by PCR, and the insertion and deletion alleles were separated and visualized by electrophoresis in a 3% agarose gel pre-stained with GelRed™ Nucleic Acid Gel Stain (BIOTIUM).

*MDM2* SNP309 genotypes were extracted from previously published data from the same individuals [11, 21, 25, 31, 32].

### Statistical analysis

Since we, and others, previously have shown the *MDM2* del1518 to be in strong linkage disequilibrium with the *MDM2* SNP309 (the del1518 del-allele being linked to the SNP309T-allele) [24, 25], we followed an analysis plan specified up-front of statistical assessments. First we performed overall risk assessments including all EC and OC patients. Then we performed sub-analyses, stratifying the data according to histology and according to SNP309-status before assessing putative associations between del1518 and cancer risk.

Possible associations between del1518 and cancer risk, both in total and in stratified groups, were evaluated by Odds Ratios (OR) and Fisher’s exact tests. ORs are given with 95% confidence intervals (CI), and *p*-values are given as two-sided. *P*-values from Fisher’s exact tests are given as two-sided and cumulative.

All statistical analyses were performed using the IBM SPSS statistics (version 22) software package.

## Results

### Distribution of del1518

The genotype distribution of the del1518 ins/del variant among the healthy controls was found to be in Hardy-Weinberg equilibrium (*p* > 0.8), with a minor allele frequency (MAF) of 0.43 (genotypes: del1518 ins/ins = 34.0%; del1518 ins/del = 46.9% and del1519 del/del = 19.2%).

An overview of the del1518 genotype distribution in the healthy controls and the two cancer types are summarized in Table 1. Among the healthy controls, the del1518 del allele was observed in individuals carrying the SNP309TT and 309TG genotypes only. Amongst the patients we found one ovarian cancer patient harboring the del1518 del/del – SNP309TG genotype and two patients (one diagnosed with endometrial- and one diagnosed with ovarian cancer) harboring the del1518 ins/del – SNP309GG genotype. Thus, del1518 was found to be in strong linkage disequilibrium (LD) with SNP309 (D’ = 0.997 for endometrial cancer cases and D’ = 0.994 for ovarian cancer cases) across all groups of individuals. Given that the SNP285C-variant is linked to the SNP309G-allele, we here, in line with previous observations [25], found no evidence of alleles carrying both SNP285C and del1518 del.

**Table 1:** *MDM2* del1518 distribution and cancer risk (OR)

Cases/controls	Genotype del1518 n (%)			OR (95% CI) del1518	Fisher exact	OR (95% CI) del1518	Fisher exact
	ins/ins	ins/del	del/del	Dominant model^a^		Recessive model^b^	
Healthy Controls	636 (34.0)	877 (46.9)	359 (19.2)	1.00	-	1.00	-
Ovarian cancer	484 (35.0)	655 (47.3)	246 (17.7)	0.96 (0.83–1.11)	0.576	0.91 (0.76–1.09)	0.316
Endometrial cancer	492 (35.0)	664 (47.3)	248 (17.7)	0.95 (0.83–1.10)	0.528	0.90 (0.76–1.08)	0.276

^a^del/del + ins/del *versus* ins/ins

^b^del/del *versus* ins/del + ins/ins

### *MDM2* del1518 and risk of endometrial – and ovarian cancer

**Fig. 1:**
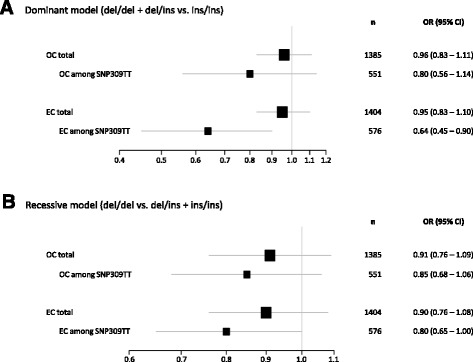
*MDM2* del1518 and impact on risk of ovarian and endometrial cancer. Forest plots showing the impact of *MDM2* del1518, in the total sample sets, and within the subgroup of SNP309, on ovarian (OC) and endometrial (EC) cancer risk. **a** dominant model and **b** recessive model

In order to evaluate the potential impact of *MDM2* del1518 status on endometrial and ovarian cancer risk, we evaluated the OR by comparing the frequency of the *MDM2* del1518 genotypes observed in ovarian and endometrial cancer cases to the SNP status among the 1,872 healthy female controls. No significant association between *MDM2* del1518 and risk of either of the two cancer types was found whether applying the dominant or the recessive model (Table 1; Fig. 1a and b).

Next, we stratified the endometrial malignant lesions and ovarian cancers into histological subgroups (endometrioid, adenosquamous, clear cell, serous papillary, hyperplasia, carcinosarcoma and undifferentiated/other types endometrial cancer; high-grade serous ovarian cancer [HGSOC], low-grade ovarian cancer [LGSOC], clear cell ovarian cancer, endometrioid ovarian cancer and mucinous ovarian cancer). No associations between *MDM2* del1518 ins/del distribution and cancer risk were observed in either of these subgroups (Additional file 1: Tables S1 and Additional file 2: Table S2).

### Impact of *MDM2* del1518 status within SNP309 genotype subgroups

Given that the *MDM2* del1518 del allele and the SNP309T was found to be in strong LD forming a distinct del1518del/SNP309T haplotype, we performed refined analyses, restricting our OR estimates to individuals carrying the SNP309TT or SNP309TG genotypes. Within the subgroup of individuals carrying the SNP309TT genotype, we observed an association between the del1518 del allele and a reduced risk of endometrial cancer, both when applying the dominant (OR = 0.64, CI = 0.45 – 0.90) and the recessive model (OR = 0.80, CI = 0.65 – 1.00; Table 2; Fig. 1a and b). In contrast, no significant association was observed among the ovarian cancer patients. Among individuals harboring the SNP309TG genotype, no association to cancer risk was recorded in either of the two cancer forms (Table 3).

**Table 2:** *MDM2* del1518 among SNP309TT

Cases/controls	Genotype del1518 n (%)			OR (95% CI) del1518	Fisher exact	OR (95% CI) del1518	Fisher exact
	ins/ins	ins/del	del/del	Dominant model^a^		Recessive model^b^	
Healthy Controls	70 (9.5)	311 (42.0)	359 (48.5)	1.00	-	1.00	
Ovarian cancer	64 (11.6)	242 (44.0)	245 (44.5)	0.80 (0.56–1.14)	0.231	0.85 (0.68–1.06)	0.159
Endometrial cancer	81 (14.1)	247 (42.9)	248 (43.1)	0.64 (0.45–0.90)	0.011	0.80 (0.65–1.00)	0.051

^a^del/del + ins/del *versus* ins/ins

^b^del/del *versus* ins/del + ins/ins

**Table 3:** *MDM2* del1518 among SNP309TG

Cases/controls	Genotype del1518 n (%)			OR (95% CI) del1518	Fisher exact
	ins/ins	ins/del	del/del^a^	Ins/del vs. ins/ins	
Healthy Controls	312 (35.5)	566 (64.5)	-	1.00	-
Ovarian cancer	213 (34.1)	412 (65.9)	-	1.07 (0.86–1.32)	0.583
Endometrial cancer	226 (35.2)	416 (64.8)	-	1.02 (0.82–1.26)	0.914

^a^No observations since del1518 del is linked to SNP309T

The majority of the endometrial cancer patients had tumors of endometroid histology. Within this subgroup (endometroid histology and SNP309TT-genotype), we found the del1518 del allele to be associated with reduced cancer risk, similar to the reduction observed for the total EC-sample set (OR = 0.64, CI = 0.44 – 0.92; dominant model; Additional file 3: Table S3). Regarding the other histology subgroups, these were too small for formal statistical risk analysis among individuals harboring the SNP309TT genotype (pooled data for these subgroups shown in Additional file 3: Table S3).

## Discussion

Previous studies have revealed *MDM2* promoter P2 SNP variants like SNP309T > G and SNP285G > C to modulate gene transcription and affect cancer risk [10, 11].

In the present study, we explored the impact of a third *MDM2* polymorphism, del1518 ins/del, located in promoter P1, on the risk of endometrial and ovarian cancer in a Caucasian population. Similar to what has been previously reported in Han Chinese [24], we confirmed del1518del/ins to be in strong linkage disequilibrium with the SNP309, forming a distinct *MDM2* del1518del/SNP309T haplotype [24, 25] with a MAF different from Chinese (MAF = 0.30) [24].

We did not detect an effect of del1518 status on the risk of endometrial or ovarian cancer in the general population. Howevver, based on previous findings indicating different *MDM2* promoter SNPs may act in concert [11] we performed subgroup analyses stratifying individuals with respect to SNP309 genotypes. (Linkage disequilibrium precluded assessment of SNP285 subgroups since all SNP285C-alleles are linked to SNP309G-alleles while del1518 del locates to the SNP309T-allele [23]).

Doing so, we found the del1518 del- allele to be associated with a reduced risk of endometrial but not for ovarian cancer applying a dominant as well as recessive model among individuals harboring the SNP309TT genotype (risk reduction of 36% and 20%, respectively). This finding is in line with previous in vitro luciferase gene reporter assay findings, showing the presence of the del1518 del allele to result in complete abrogation of promoter activity [12]. Lack of effect on ovarian cancer risk is also in accordance with the results from a previous study performed in Chinese individuals [28]. However, other studies have found the del1518 del allele to confer an *increased* risk for other cancer forms like hepatocellular carcinomas [26] and left sided colon cancer among individuals carrying the SNP309TG genotype [25], as well as increased risk for uterine leiomyomas (non-cancerous growths in the uterus) [27], but no associations to epithelial ovarian cancer, esophageal squamous cell carcinoma, breast, lung or prostate cancer risk [24, 25, 28–30]. Taken together, these findings suggests the del 1518 ins/del variant may have different effects on risk for cancer development in different organs, partly dependent on interactions with other *MDM2* SNP variants, similar to what has been observed for promoter variants like SNP309 and SNP285 [11, 17, 21, 32]. Interestingly, both endometrial and ovarian cancer risk has previously been found to be reduced by the SNP285C-allele [11, 21]. Further, the SNP309G-allele has been found to be associated with an increased but also a reduced cancer risk across different tissues [16, 18, 34, 35]. Also the fact that mice carrying the human *MDM2* SNP309G allele only displayed elevated *MDM2* expression in a few tissues [36] supports the hypothesis that SNP309 may act as a cancer risk factor in distinct tissues only. Thus, our observation of del1518 del variant reducing the risk of endometrial but not ovarian cancer may indicate that this variant is modulating the binding of transcription factors that are differentially expressed in these two tissues.

Given that we previously have found a SNP in the *MDM4* 3 ‘UTR (SNP34091) to be associated with increased risk of serous, and in particular high grade serous ovarian cancer [31], we performed subgroup analysis based on histology status with respect to del1581. These subgroup analyses did not reveal any histology specific effects of del1518 status, indicating that this variant have no effect on tumor progression.

## Conclusion

In conclusion we find the *MDM2* SNP del1518 del variant to be associated with reduced risk of endometrial cancer among individuals carrying the SNP309TT genotype, an observation warranting confirmation in independent studies.

## Additional files

Additional file 1: Table S1. MDM2 del158 genotype distribution in OC subgroups. (DOCX 17 kb)
Additional file 2: Table S2. MDM2 del158 genotype distribution in EC subgroups. (DOCX 17 kb)
Additional file 3: Table S3. MDM2 del158 genotype distribution among individuals with SNP309TT genitype in EC subgroups. (DOCX 15 kb)
Additional file 4: Table S4. Raw data; genotypes for all individuals in study. (XLSX 118 kb)

## Abbreviations

CI: Confidence intervals
CONOR: Cohort of Norway
EC: Endometrial cancer
HGSOC: High grade serous ovarian cancer
LD: Linkage disequilibrium
LGSOC: Low grade serous ovarian cancer
MDM2: Mouse Double Minute 2
OC: Ovarian cancer
OR: Odds Ratios
SNP: Single nucleotide polymorphism
Sp1: Specificity protein 1
