# Supplementary material for: MDM2 promoter polymorphism del1518 (rs3730485) and its impact on endometrial and ovarian cancer risk
Source: BMC Cancer. 2017 Feb 3;17:97. doi: 10.1186/s12885-017-3094-y (PMC5291962; doi:10.1186/s12885-017-3094-y)
Supplement: Additional file 1: Table S1. — MDM2 del158 genotype distribution in OC subgroups. (DOCX 17 kb) [file 12885_2017_3094_MOESM1_ESM.docx]

| **Table S1. *MDM2* del1518 distribution and cancer risk (OR) in subgroups of ovarian cancer.** | | | | | | | | | | | | |
| --- | --- | --- | --- | --- | --- | --- | --- | --- | --- | --- | --- | --- |
| **Cases/** | **Genotype** | | |  | **OR (95% CI)** |  | **Fisher** |  | **OR (95% CI)** |  | **Fisher** |  |
| **controls** | **del1518 n (%)** | | |  | **del1518** |  | **exact** |  | **del1518** |  | **exact** |  |
|  | **ins/ins** | **ins/del** | **del/del** |  | **Dominant model^a^** |  |  |  | **Recessive model^b^** |  |  |  |
| **Healthy**  **Controls** | 636 (34.0) | 877 (46.9) | 359 (19.2) |  | 1.00 |  | - |  | 1.00 |  | - |  |
|  |  |  |  |  |  |  |  |  |  |  |  |  |
| **Serous ovarian**  **cancer** | 306 (33.9) | 441 (48.8) | 156 (17.3) |  | 1.04 (0.85-1.19) |  | 1.000 |  | 0.88 (0.72-1.08) |  | 0.231 |  |
|  |  |  |  |  |  |  |  |  |  |  |  |  |
| **LGSOC^*^** | 119 (34.2) | 168 (48.3) | 61 (17.5) |  | 0.99 (0.78-1.26) |  | 0.951 |  | 0.90 (0.66-1.21) |  | 0.503 |  |
|  |  |  |  |  |  |  |  |  |  |  |  |  |
| **HGSOC^*^** | 164 (35.0) | 227 (48.4) | 78 (16.6) |  | 0.96 (0.77-1.18) |  | 0.703 |  | 0.84 (0.64-1.10) |  | 0.233 |  |
|  |  |  |  |  |  |  |  |  |  |  |  |  |
| **Clear cell ovarian cancer** | 22 (32.8) | 31 (46.3) | 14 (20.9) |  | 1.05 (0.63-1.77) |  | 0.896 |  | 1.11 (0.61-2.03) |  | 0.752 |  |
|  |  |  |  |  |  |  |  |  |  |  |  |  |
| **Endometrioid Ovarian cancer** | 52 (39.4) | 63 (47.7) | 17 (12.9) |  | 0.79 (0.55-1.14) |  | 0.218 |  | 0.62 (0.37-1.05) |  | 0.083 |  |
|  |  |  |  |  |  |  |  |  |  |  |  |  |
| **Mucinous ovarian cancer** | 22 (40.0) | 22 (40.0) | 11 (20.0) |  | 0.77 (0.45-1.34) |  | 0.387 |  | 1.55 (0.54-2.06) |  | 0.862 |  |
|  |  |  |  |  |  |  |  |  |  |  |  |  |

^a^ del/del + ins/del versus ins/ins

^b^ del/del versus ins/del + ins/ins

^*^ Grade information lacking for some of the serous ovarian cancer patients. I.e. the number of LGSOC + HGSOC does not match the total number if serous OCs
