# Supplementary material for: MDM2 promoter polymorphism del1518 (rs3730485) and its impact on endometrial and ovarian cancer risk
Source: BMC Cancer. 2017 Feb 3;17:97. doi: 10.1186/s12885-017-3094-y (PMC5291962; doi:10.1186/s12885-017-3094-y)
Supplement: Additional file 2: Table S2. — MDM2 del158 genotype distribution in EC subgroups. (DOCX 17 kb) [file 12885_2017_3094_MOESM2_ESM.docx]

| **Table S2. *MDM2* del1518 distribution and cancer risk (OR) in subgroups of endometrial cancer.** | | | | | | | | | | | | |
| --- | --- | --- | --- | --- | --- | --- | --- | --- | --- | --- | --- | --- |
| **Cases/** | **Genotype** | | |  | **OR (95% CI)** |  | **Fisher** |  | **OR (95% CI)** |  | **Fisher** |  |
| **controls** | **del1518 n (%)** | | |  | **del1518** |  | **exact** |  | **del1518** |  | **exact** |  |
|  | **ins/ins** | **ins/del** | **del/del** |  | **Dominant model^a^** |  |  |  | **Recessive model^b^** |  |  |  |
| **Healthy**  **Controls** | 636 (34.0) | 877 (46.9) | 359 (19.2) |  | 1.00 |  | - |  | 1.00 |  | - |  |
|  |  |  |  |  |  |  |  |  |  |  |  |  |
| **Endometrioid** | 379 (35.6) | 494 (46.4) | 192 (18.0) |  | 0.93 (0.80-1.09) |  | 0.397 |  | 0.93 (0.76-1.13) |  | 0.461 |  |
|  |  |  |  |  |  |  |  |  |  |  |  |  |
| **Adenosquamos** | 1 (9.1) | 7 (63.6) | 3 (27.3) |  | na |  | na |  | na |  | na |  |
|  |  |  |  |  |  |  |  |  |  |  |  |  |
| **Clear cell** | 15 (35.7) | 21 (50.0) | 6 (14.3) |  | 0.93 (0.49-1.75) |  | 0.869 |  | 0.70 (0.29-1.68) |  | 0.552 |  |
|  |  |  |  |  |  |  |  |  |  |  |  |  |
| **Serous papillary** | 39 (32.0) | 60 (49.2) | 23 (18.9) |  | 1.10 (0.74-1.62) |  | 0.694 |  | 0.98 (0.61-1.56) |  | 1.000 |  |
|  |  |  |  |  |  |  |  |  |  |  |  |  |
| **Hyperplasia** | 0 | 4 | 0 |  | na |  | na |  | na |  | na |  |
|  |  |  |  |  |  |  |  |  |  |  |  |  |
| **Carcinosarcoma** | 18 (34.0) | 28 (52.8) | 7 (13.2) |  | 1.00 (0.56-1.78) |  | 1.000 |  | 0.64 (0.29-1.43) |  | 0.374 |  |
|  |  |  |  |  |  |  |  |  |  |  |  |  |
| **Undiff/other** | 8 (34.8) | 10 (43.5) | 5 (21.7) |  | 0.97 (0.41-2.29) |  | 1.000 |  | 1.17 (0.43-3.17) |  | 0.789 |  |
|  |  |  |  |  |  |  |  |  |  |  |  |  |

^a^ del/del + ins/del versus ins/ins

^b^ del/del versus ins/del + ins/ins
