# Supplementary material for: MDM2 promoter polymorphism del1518 (rs3730485) and its impact on endometrial and ovarian cancer risk
Source: BMC Cancer. 2017 Feb 3;17:97. doi: 10.1186/s12885-017-3094-y (PMC5291962; doi:10.1186/s12885-017-3094-y)
Supplement: Additional file 3: Table S3. — MDM2 del158 genotype distribution among individuals with SNP309TT genitype in EC subgroups. (DOCX 15 kb) [file 12885_2017_3094_MOESM3_ESM.docx]

| **Table S3. *MDM2* del1518 among SNP309TT and cancer risk in subgroups of endometrial cancer** | | | | | | | | | | | |
| --- | --- | --- | --- | --- | --- | --- | --- | --- | --- | --- | --- |
| **Cases/** | **Genotype** | | |  | **OR (95% CI)** |  | **Fisher** |  | **OR (95% CI)** |  | **Fisher** |
| **controls** | **del1518 n (%)** | | |  | **del1518** |  | **exact** |  | **del1518** |  | **exact** |
|  | **ins/ins** | **ins/del** | **del/del** |  | **Dominant model^a^** |  |  |  | **Recessive model^b^** |  |  |
| **Healthy**  **Controls** | 70 (9.5) | 311 (42.0) | 359 (48.5) |  | 1.00 |  | - |  | 1.00 |  |  |
|  |  |  |  |  |  |  |  |  |  |  |  |
| **Endometroid** | 61 (14.1) | 181 (41.7) | 192 (44.2) |  | 0.63 (0.44-0.92) |  | 0.021 |  | 0.84 (0.66-1.07) |  | 0.164 |
|  |  |  |  |  |  |  |  |  |  |  |  |
| **Other^*^** | 14 (13.1) | 49 (45.8) | 44 (41.1) |  | 0.70 (0.38-1.28) |  | 0.290 |  | 0.74 (0.49-1.12) |  | 0.178 |
|  |  |  |  |  |  |  |  |  |  |  |  |

^a^ del/del + ins/del versus ins/ins

^b^ del/del versus ins/del + ins/ins

* Due to small numbers the other endometrial histological classes were analyzed as one group, within individuals with the SNP309TT genotype.
